# Acute urinary retention in a pre-school girl with constipation

**DOI:** 10.1016/j.rpped.2015.03.007

**Published:** 2015

**Authors:** Guillermo A. Ariza Traslaviña, Luiz Antonio Del Ciampo, Ivan Savioli Ferraz

**Affiliations:** a Hospital das Clínicas, Faculdade de Medicina de Ribeirão Preto, Universidade de São Paulo (FMRP-USP), Ribeirão Preto, SP, Brazil; b Department of Child Care and Pediatrics, Faculdade de Medicina de Ribeirão Preto, Universidade de São Paulo (FMRP-USP), Ribeirão Preto, SP, Brazil

**Keywords:** Urinary retention, Constipation, Child

## Abstract

**Objective:**

To report a case of a preschool girl who developed acute urinary retention associated with constipation.

**Case description:**

A girl aged six years old presented a 24 h history of inability to urinate. She was went twice to the emergency room during this period. In the first admission, 12 h after the onset of the symptoms, she presented abdominal pain and acute urinary retention. After the drainage by urinary catheterization of 300 mL of clear urine, she presented relief of the symptoms and, as urinalysis had no change, the patient was discharged home. Twelve hours after the first visit, she returned to the emergency room complaining about the same symptoms. At physical examination, there was only a palpable and distended bladder up to the umbilicus with no other abnormalities. Again, a urinary catheterization was performed, which drained 450 mL of clear urine, with immediate relief of the symptoms. Urinalysis and urine culture had no abnormalities. During the anamnesis, the diagnosis of constipation was considered and a plain abdominal radiography was performed, which identified large amount of feces throughout the colon (fecal retention). An enema with a 12% glycerin solution was prescribed for three days. During follow-up, the child used laxatives and dietary modifications, this contributed to the resolution of the constipation. There were no other episodes of urinary retention after 6 months of follow-up.

**Comments:**

Acute urinary retention in children is a rare phenomenon and constipation should be considered as a cause.

## Introduction

Acute urinary retention is defined as the incapacity to voluntarily urinate for more than 12 h, despite the presence of an intravesical urine volume higher than that expected for age [(age in years + 2) × 30 mL]^1^ or the presence of a distended bladder on physical examination. It is a common symptom in the adult male population, mainly due to benign prostatic hyperplasia,^2^ whereas its presentation is rare in children, being associated to neurological diseases, infections in the urinary tract and other sites, severe voiding dysfunction, side effects of some drugs (especially anticholinergics), tumors, anatomical and emotional problems, as well as trauma.^3^^-^ 5 Although mentioned in some studies, constipation does not appear among the most common causes of acute urinary retention.^3^^-^ 5 Although the prevalence of intestinal constipation in our pediatric population is high,^6^ the report of its association with urinary retention in the Brazilian medical literature is rare. Therefore, the aim of this article is to present the case of a six-year-old child with acute urinary retention and constipation, aiming to expand the possibilities for differential diagnosis and alert pediatricians at the initial evaluation of these patients.

## Case report

A female child, aged six years old, born to non-consanguineous parents, with an ectopic left kidney (pelvic) and normal kidney function, came for the second time to the emergency department of a district health unit in the Ribeirão Preto city (state of São Paulo) showing irritability, generalized abdominal pain of moderate intensity and incapacity to release the bladder sphincter for 24 h. According to the mother, the child had no prior voiding disorder and did not use any medication, having been treated 12 h before at the same emergency department with similar complaints. At the first consultation, an increase in bladder volume was observed and urinary catheterization was performed, with 300 mL output of clear urine, followed by immediate abdominal pain improvement. On that occasion, a urinalysis test was requested, which showed no alterations, and the child was discharged home. However, the symptoms had reappeared in the last 12 h and the child was once again brought to the emergency department.

At the second consultation, the child was afebrile, weighed 18 kg, had a respiratory rate of 20 breaths per minute, heart rate of 90 beats per minute and blood pressure of 90/60 mmHg, and was between the 25th and 50th percentiles for height/age index by gender. Additionally, she presented with pain on palpation of the lower abdomen and shifting dullness in the hypogastric region, where a mass of cystic consistency was palpable, compatible with bladder distention, which reached the umbilicus. There were no alterations in the vulvovaginal area. New bladder decompression was performed through catheterization, with a 450 mL output of clear urine, followed once again by marked pain relief after the procedure. Urine samples were obtained for urine culture and urinalysis, which showed no alterations.

**Figure 1:**
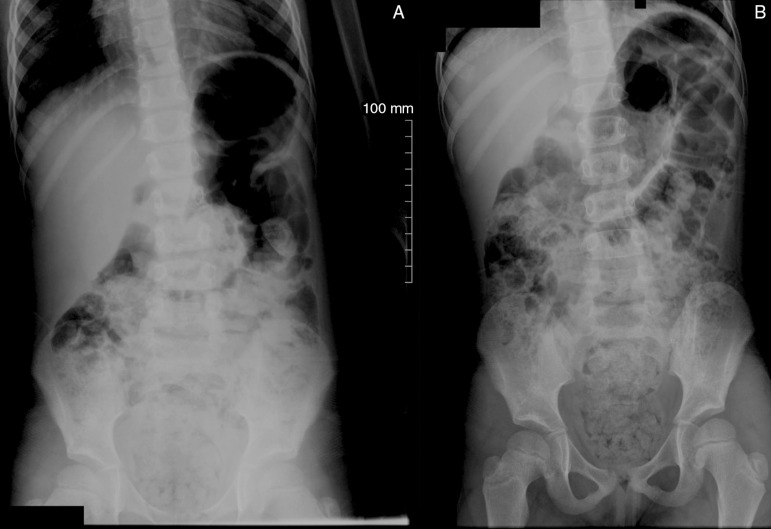
Abdominal X-rays in the standing (A) and supine (B) position, disclosing large amount of stool in the ascending and descending colon, sigmoid and rectum. Dilation in the rectosigmoid segment, secondary to the presence of stool, can also be observed.

During the anamnesis, we obtained the information that the child had daily bowel habits with hard, dry and thick stools for at least three years, occasionally using oral laxatives without medical advice; she had received a diagnosis of intestinal constipation two years before in a Primary Care Center, but adherence to the dietary guidelines was not adequate (there was no mention of laxative prescription). Considering these facts, a plain abdominal radiograph was performed in the anteroposterior view (AP) in the supine and standing positions (Fig. 1), which disclosed images compatible with the presence of stool in the rectum, cecum and along the colon, in addition to fecal impaction (retention) and dilation in the rectal ampulla.

While still at the emergency department, she was submitted to fecal disimpaction with a 12% glycerin enema solution and this treatment was maintained for 3 consecutive days. Mineral oil (1 mL/kg/day orally, divided into two doses) was prescribed for continuous use at home. The child's mother received recommendations for modification of the child's eating habits, aiming mainly at increasing the intake of water and fiber-rich foods. Stool samples for parasitological examination were also requested.

The child returned for consultation at the outpatient clinic of the Primary Care Center eight days after symptom onset, being in good health status, without abdominal pain, maintaining adequate diuresis and showing no relevant findings on physical examination. At this time, the results of urine culture and parasitological tests were verified, which were negative.

During a six-month follow up, the child did not have new episodes of acute urinary retention. She was regularly followed at the Primary Care Center, showing improvement in the eating habits (increased fiber and water intake) and evacuating twice daily without difficulty, with soft and thinner stools. Considering the improvement of the child's constipation, the use of mineral oil was suspended after three months.

## Discussion

Acute urinary retention is a relatively uncommon phenomenon in children. We report on a case of acute urinary retention in a six-year-old female child who, concomitantly, had chronic constipation and a pelvic kidney to the left.

Despite the high prevalence of chronic constipation in children, acute urinary retention episodes associated with this morbidity are rarely mentioned in the international and Brazilian medical literature. In a survey carried out in an American hospital from 1993 to 2000, 53 patients aged six months to 17 years old were identified.

The following were identified as the most common causes of acute urinary retention in decreasing order of frequency: neurological disorders (17%), severe voiding disorders (15%), urinary tract infections (13%), constipation (13%), and side effects of medications (13%). Additionally, 2% of patients showed an association of urinary tract infection and constipation as cause of acute urinary retention, thus comprising 15% of frequency for each of these entities. The boys were more affected than girls, and the mean age in the respective genders was 5 and 4 years.^3^ In another survey carried out in three Iranian hospitals from 1996 to 2003, the authors found 86 children aged up to 14 years with acute urinary retention, of which main causes were lower urinary tract stones (28%), neurological disorders (10%) and local trauma (10%). Constipation showed to be an uncommon cause, being observed in only 5% of patients.^4^ In both abovementioned surveys, individuals in the immediate postoperative period using opioids, those with mental retardation and chronic neurological disorders, and immobile ones were excluded. In a more recent survey, carried out in a tertiary hospital in Israel, 56 patients younger than 18 years treated for acute urinary retention between the years 2000 and 2012 were found. In these patients, the most common causes of acute urinary retention were: mechanical obstruction (25%), infectious or inflammatory processes (18%) and fecal impaction (13%).^5^ In the latter study, in addition to newborns, all individuals in the immediate postoperative period, those submitted to urethral procedures and those with neurological disorders were excluded. In a Brazilian study performed with 163 children with chronic functional constipation followed in a pediatric gastroenterology clinic in Botucatu city (state of São Paulo), there was a prevalence of 8.6% of urinary retention as a complication of the baseline medical condition. It is noteworthy that in the latter study, 43.4% of the children had one or more urinary alterations (enuresis, infections and urinary retention outbreaks) associated with constipation.^7^

Regarding the patient described in this case report, the only alteration found was the large amount of stool observed in the plain abdominal radiography. This examination - when performed in the AP view, in the supine and standing positions, and even though it does not establish the diagnosis of constipation -, allows, assessing the presence of fecal retention and possible abdominal or pelvic masses or even calcifications in the urinary tract that could help explain the acute urinary retention. Symptom disappearance with bowel habit improvement and decreased stool consistency indicate that chronic constipation was the cause of the acute urinary retention in this patient. This child had a once-daily bowel habit with hard, dry and thick stools for at least three years and was undergoing an irregular follow-up at a Primary Care Center with a diagnosis of constipation for two years. The mother had received dietary recommendations to increase the child's intake of fiber and water, and she occasionally gave the child laxatives. The delay in seeking medical care, poor adherence to the treatment plan and prescription of inadequate treatments are commonly reported in the literature regarding constipation.^8^^,^ 9 The low morbidity of the initial picture of constipation, lack of knowledge regarding the children's normal pattern of evacuation by their parents, lack of an individualized nutritional plan and the prescription of unpalatable laxatives may explain poor treatment adherence.^8^^,^ 10 ^-^ 12 The absence of a correct approach to the treatment of constipation can lead to disease complications, as described in the child in our case report.^8^

The association of constipation with urinary disorders is well established in the medical literature.^13^^,^ 14 In the pediatric population, several studies have shown a strong association between constipation and the presence of urinary disorders, such as incontinence and urinary urgency^15^^,^ 16; additionally, larger volumes of post-micturition residual urine and urinary tract dilation, including the ureteropelvic tract, have been most commonly observed in constipated children.^17^^,^ 18 Thus, acute urinary retention probably constitutes one of the clinical presentations of incomplete emptying of the bladder in children with constipation. The physiopathology of the association between constipation and voiding disorders could be explained by several factors. The bladder and the rectum share a close embryological association (cloaca) during the pelvic floor formation, sharing the same innervation, nerve roots S2 to S4, which control motor function of the internal anal and urinary sphincters.^13^^,^ 18 Experiments with rats found that rectal distention with a balloon diminished bladder contractility.^19^ Chronic retention could also lead to involuntary contraction of the pelvic floor muscles and the external anal sphincter, making bladder emptying difficult.^14^^,^ 18Additionally, considering the close anatomical association, the presence of impacted stool in the rectum reduces bladder functional capacity, resulting in a feeling of earlier bladder emptying.^14^ Moreover, a chronically full rectal ampulla can lead to vesical trigone irritation, invaginations in the posterior wall of the bladder and urethral obstruction.^20^

The child whose case was reported in this paper had an ectopic kidney to the left. The literature has shown few reports of acute urinary retention episodes in individuals with genitourinary tract deformities, such as malformations of the female (didelphic uterus and imperforate hymen)^21^^,^ 22 and male genital tracts (seminal vesicle cyst)^23^ associated with unilateral renal agenesis; however, there are no reported cases involving a picture of acute urinary retention with ectopic kidney. In the authors’ opinion, the patient's ectopic kidney described here did not contribute in any way to the acute urinary retention.

It can be concluded that acute urinary retention is a rare event in the pediatric age group. Therefore, although it is not one of the most common causes, intestinal constipation, given its high prevalence, should be considered when treating children and adolescents with acute urinary retention.
