# Supplementary material for: Acute urinary retention in a pre-school girl with constipation
Source: Rev Paul Pediatr. 2015 Oct-Dec;33(4):488–92. doi: 10.1016/j.rpped.2015.03.007 (PMC4685571; doi:10.1016/j.rpped.2015.03.007)
Supplement: Translation

# Translation: Retenção urinária aguda em pré-escolar feminina com constipação intestinal

**Authors:** Guillermo A. Ariza Traslaviña, Luiz Antonio Del Ciampo, Ivan Savioli Ferraz

**Affiliations:** a Hospital das Clínicas, Faculdade de Medicina de Ribeirão Preto, Universidade de São Paulo (FMRP-USP), Ribeirão Preto, SP, Brasil; b Departamento de Puericultura e Pediatria, Faculdade de Medicina de Ribeirão Preto, Universidade de São Paulo (FMRP-USP), Ribeirão Preto, SP, Brasil

**Keywords:** Retenção urinária, Constipação intestinal, Criança

## Abstract

**Objetivo:**

Relatar um caso de criança que desenvolveu retenção urinária aguda associada à constipação intestinal.

**Descrição do caso:**

Menina, seis anos, havia 24 horas apresentara incapacidade de liberação de esfíncter vesical. Foi atendida por duas vezes em um serviço de emergência nesse período. Na primeira consulta, 12 horas após o início do quadro, apresentava dor abdominal e retenção urinária aguda e foi feita sondagem de alívio com saída de 300mL de urina clara. Houve alívio imediato dos sintomas e, como o exame de urina tipo 1 não apresentou alterações, a paciente recebeu alta. No segundo atendimento, 12 horas após a primeira consulta, apresentava as mesmas queixas. Ao exame físico, observou-se apenas bexiga palpável e distendida até a cicatriz umbilical, sem outras alterações. Nova sondagem vesical foi feita com saída de 450mL de urina clara, com alívio imediato dos sintomas. Nenhuma anormalidade foi observada no exame de urina tipo 1 e na urocultura. Durante a anamnese, foi levantada a hipótese diagnóstica de constipação intestinal. Foi feita radiografia simples de abdome, que identificou grande quantidade de fezes em todo o cólon (retenção fecal). Enema com solução glicerinada a 12% foi prescrito por três dias. Durante o seguimento a criança fez uso de laxativos e modificações na dieta que contribuíram para a resolução da constipação intestinal. Não houve repetição do quadro de retenção urinária aguda após seis meses de acompanhamento.

**Comentários:**

A retenção urinária aguda em crianças é um fenômeno raro e a constipação intestinal deve ser considerada como uma das causas.

## Introdução

A retenção urinária aguda é definida como a incapacidade de urinar voluntariamente por mais de 12 horas, apesar de um volume urinário intravesical maior do que esperado pela idade [(idade em anos+2)×30mL]^1^ ou pela presença de uma bexiga distendida ao exame físico. Trata-se de um sintoma comum na população adulta masculina, devido, principalmente, à hiperplasia prostática benigna,^2^ enquanto sua apresentação é rara na população pediátrica e está relacionada com doenças neurológicas, infecções do trato urinário e de outros sítios, disfunções miccionais graves, efeitos colaterais de algumas drogas (em especial, as anticolinérgicas), tumores, problemas anatômicos, emocionais e traumatismos.^3^^-^ 5 Apesar de também citada em alguns estudos, a constipação intestinal não aparece entre as causas mais comuns de retenção urinária aguda.^3^^-^ 5 Embora a prevalência de constipação intestinal na nossa população infantil seja elevada,^6^ o relato de sua associação com retenção urinária na literatura médica brasileira é raro. Portanto, o objetivo deste artigo é apresentar um caso de retenção urinária aguda em uma menina de seis anos, com constipação intestinal, a fim de ampliar as possibilidades para o diagnóstico diferencial e alertar os pediatras na avaliação inicial desses pacientes.

## Descrição do caso

Criança do sexo feminino, seis anos, filha de pais não consanguíneos, portadora de rim esquerdo ectópico (pélvico) e função renal normal, comparece pela segunda vez a um serviço de emergência de uma unidade distrital de saúde na cidade de Ribeirão Preto (SP). Apresentava irritabilidade, dor abdominal generalizada de moderada intensidade e incapacidade para liberar o esfíncter vesical havia 24 horas. Segundo a mãe, a criança não apresentava distúrbio miccional prévio e não fazia uso de medicamento algum. Já comparecera havia 12 horas ao mesmo serviço de emergência com queixas semelhantes. Nessa primeira visita foi constatado um aumento de volume da bexiga e feita sondagem vesical de alívio com saída de 300mL de urina clara, com melhoria imediata da dor abdominal. Nessa ocasião foi solicitado um exame de urina tipo 1, que não apresentou alterações, e a criança foi liberada para casa. Entretanto, nas últimas 12 horas os sintomas reapareceram e a menina foi novamente levada ao serviço de emergência.

No segundo atendimento, a criança encontrava-se afebril, com peso de 18kg, frequência respiratória de 20 incursões por minuto, frequência cardíaca de 90 batimentos por minuto e pressão arterial de 90/60mmHg. Estava entre os percentis 25 e 50 para o índice estatura/idade segundo o sexo. Além disso, apresentava dor à palpação no andar inferior do abdome e macicez na região hipogástrica, onde se palpava uma massa de consistência cística, compatível com distensão vesical, que chegava até a altura da cicatriz umbilical. Não havia alterações na região vulvovaginal. Foi feita nova descompressão da bexiga por meio de sondagem vesical, observou-se a saída de 450mL de urina clara e houve, novamente, melhoria drástica da dor após esse procedimento. Amostras de urina foram obtidas para urocultura e exame de urina tipo 1, que não apresentaram alterações.

Durante a anamnese, obteve-se a informação de que a criança apresentava hábito intestinal diário com fezes ressecadas, calibrosas e em cíbalos havia pelo menos três anos. Fazia uso ocasional de laxantes por via oral sem orientação médica; recebera diagnóstico de constipação intestinal havia dois anos em unidade básica de saúde, mas a adesão às orientações alimentares não foi adequada (não havia menção de prescrição médica de laxantes). Diante disso, foi feita radiografia simples de abdome, nas incidências anteroposterior (AP) em decúbito dorsal e na posição ortostática (Fig. 1), que evidenciou imagens compatíveis com fezes em reto, ceco e ao longo de todo o cólon, além de dilatação e impactação (retenção) de fezes em ampola retal.

**Figura 1:** Radiografias de abdome em posição ortostática (A) e decúbito dorsal (B) em que se observa grande quantidade de fezes nos cólons ascendente e descendente, sigmoide e reto. Nota-se também dilatação do segmento reto sigmoide secundário à presença das fezes.

Ainda no serviço de emergência, procedeu-se à desimpactação fecal com enema de solução glicerinada a 12%. Esse tratamento foi mantido por três dias consecutivos. Óleo mineral (1mL/kg/dia por via oral, dividido em duas tomadas) foi prescrito para uso contínuo em casa. A mãe da criança recebeu orientações para a modificação dos hábitos alimentares da criança, com vistas, principalmente, ao aumento da ingestão de água e alimentos ricos em fibras. Também foram solicitadas amostras de fezes para exame parasitológico.

A criança foi atendida oito dias após o início do quadro no ambulatório de sua unidade básica de saúde. Apresentou-se em boas condições, sem dor abdominal, mantinha diurese adequada e sem achados relevantes ao exame físico. Nessa ocasião, foram verificados os resultados de urocultura e parasitológicos de fezes, que resultaram negativos.

Durante um seguimento de seis meses, a criança não apresentou novos episódios de retenção urinária aguda. Encontrava-se em seguimento regular na unidade básica de saúde, com melhoria dos hábitos alimentares (aumento da ingestão de fibras e água) e evacuava duas vezes ao dia, sem dificuldades, fezes pastosas e não calibrosas. Diante da melhoria do quadro de constipação intestinal da criança, o óleo mineral foi suspenso após três meses de uso.

## Discussão

A retenção urinária aguda é um fenômeno relativamente incomum em crianças. Neste artigo relata-se um caso de retenção urinária aguda em criança do sexo feminino, de seis anos, que apresentava, concomitantemente, constipação intestinal crônica funcional e rim pélvico à esquerda.

Apesar da elevada prevalência de constipação intestinal na população infantil, episódios de retenção urinária aguda associados a essa morbidade raramente são citados na literatura médica internacional e brasileira. Em levantamento feito em hospital americano de 1993 a 2000, encontraram-se 53 pacientes entre seis meses e 17 anos. Dentre as causas mais comuns de retenção urinária aguda estavam, em ordem decrescente de frequência, afecções neurológicas (17%), disfunções graves da micção (15%), infecções do trato urinário (13%), constipação intestinal (13%) e efeitos colaterais de medicamentos (13%). Além disso, 2% dos pacientes apresentaram como causa de retenção urinária aguda uma associação de infecção do trato urinário e constipação intestinal. Perfizeram-se, dessa forma, 15% de frequência para cada uma dessas entidades. Os meninos foram mais afetados do que as meninas e a média de idade nos respectivos sexos foi de cinco e quatro anos.^3^ Em outro levantamento feito em três hospitais iranianos de 1996 a 2003, os autores encontraram 86 crianças até 14 anos com retenção urinária aguda, cujas principais causas foram cálculos no trato urinário baixo (28%), afecções neurológicas (10%) e traumas locais (10%). A constipação intestinal mostrou-se uma causa pouco comum, observada em apenas 5% dos pacientes.^4^ Em ambos os levantamentos citados, indivíduos que estavam no período pós-operatório imediato, em uso de opioides ou que apresentavam retardo mental, distúrbios neurológicos crônicos e imobilidade foram excluídos do estudo. Em um levantamento mais recente, feito em um hospital terciário em Israel, foram encontrados 56 pacientes menores de 18 anos atendidos devido à retenção urinária aguda de 2000 a 2012. Nesses pacientes, as causas mais comuns de retenção urinária aguda foram: obstrução mecânica (25%), processos infecciosos ou inflamatórios (18%) e impactação fecal (13%).^5^ Nesse último estudo, além de recém-nascidos, foram excluídos os indivíduos no período pós-operatório imediato, pacientes após procedimentos uretrais e aqueles com distúrbios neurológicos. Em um estudo brasileiro feito com 163 crianças portadoras de constipação intestinal crônica funcional seguidas em um ambulatório de gastroenterologia pediátrica na cidade de Botucatu (SP), observou-se uma prevalência de 8,6% de retenção urinária como complicação do quadro clínico de base. Vale a pena ressaltar que, nesse último trabalho, 43,4% das crianças estudadas apresentavam uma ou mais alterações urinárias (enurese, infecções e surtos de retenção) associadas à constipação intestinal.^7^

Em relação ao caso da paciente descrita neste artigo, a única alteração encontrada foi a grande quantidade de fezes observada na radiografia simples de abdome. Esse exame, na incidência anteroposterior (AP) em decúbito dorsal e na posição ortostática, embora não faça o diagnóstico de constipação intestinal, permite, entre outras coisas, avaliar a presença de retenção fecal ou de eventuais massas abdominais ou pélvicas ou, ainda, de calcificações no trajeto do trato urinário que poderiam ajudar a explicar a retenção urinária aguda. O desaparecimento dos sintomas com a melhoria do hábito intestinal e a diminuição da consistência das fezes indicam que a constipação intestinal foi a causa da retenção urinária aguda nessa paciente. Essa criança já apresentava hábito intestinal diário com fezes ressecadas, calibrosas e em cíbalos havia pelo menos três anos e estava em seguimento irregular na unidade básica de saúde com o diagnóstico de constipação intestinal havia dois anos. Havia recebido orientações alimentares com o objetivo de aumentar a ingestão de fibras e de água e fazia uso ocasional de laxantes. A demora em procurar assistência médica, a má adesão ao plano terapêutico e a prescrição de tratamento inadequado são relatadas comumente na literatura em relação à constipação intestinal.^8^^,^ 9 A baixa morbidade do quadro inicial da constipação intestinal, o desconhecimento do padrão normal de evacuação de seus filhos pelos pais, a ausência de uma orientação alimentar individualizada e a prescrição de laxantes pouco palatáveis podem explicar a má adesão ao tratamento.^8^^,^ 10 ^-^ 12 A ausência de uma abordagem correta no tratamento da constipação intestinal pode levar a complicações do quadro, como o descrito na criança em questão.^8^

A associação da constipação intestinal com os distúrbios da micção é bem estabelecida na literatura médica.^13^^,^ 14 Na população pediátrica, vários estudos têm mostrado uma elevada associação de constipação intestinal com a presença de distúrbios miccionais, tais como a incontinência e a urgência urinária;^15^^,^ 16 além disso, maiores volumes residuais de urina pós-miccional e dilatação das vias urinárias, incluindo o trato pielocalicial, foram observados mais comumente em crianças constipadas.^17^^,^ 18 Assim, a retenção urinária aguda provavelmente constitui uma das apresentações clínicas de esvaziamento incompleto da bexiga em crianças portadoras de constipação intestinal. A fisiopatologia da associação entre constipação intestinal e distúrbios da micção poderia ser explicada por vários fatores. A bexiga e o reto compartilham estreita relação embriológica (cloaca) durante a formação do assoalho pélvico e a mesma inervação, as raízes nervosas de S2 a S4, as quais controlam a função motora dos esfíncteres anal interno e urinário.^13^^,^ 18 Experimentos com ratos observaram que a distensão retal com um balão diminuía a contratilidade da bexiga.^19^ A retenção crônica poderia levar, também, à contração involuntária dos músculos do assoalho pélvico e do esfíncter anal externo e dificultar o esvaziamento da bexiga.^14^^,^ 18Ainda, tendo em vista a íntima relação anatômica, a presença de fezes impactadas no reto reduz a capacidade funcional da bexiga e provoca uma sensação de esvaziamento da bexiga mais precoce.^14^Ainda, uma ampola retal cronicamente repleta pode levar à irritação do trígono vesical, invaginações na parede posterior da bexiga e obstrução uretral.^20^

A criança cujo caso foi apresentado neste artigo apresentava rim ectópico à esquerda. Na literatura há raros relatos de episódios de retenção urinária aguda em indivíduos portadores de deformidades do trato genitourinário, tais como más-formações do aparelho genital feminino (útero didelfo e hímen imperfurado)^21^^,^ 22 e masculino (cisto vesicosseminal)^23^ associados à agenesia renal unilateral; entretanto, não há casos relatados que associem o quadro de retenção urinária aguda com rim ectópico. Na opinião dos autores, o rim ectópico da paciente aqui descrita em nada contribuiu para a retenção urinária aguda.

Pode-se concluir que a retenção urinária aguda é um evento raro na faixa etária pediátrica. Dessa forma, apesar de não ser uma das causas mais comuns, a constipação intestinal, em face de sua elevada prevalência, deve ser considerada quando do atendimento de crianças e adolescentes com retenção urinária aguda.
